# KMT5B in Cancerous and Noncancerous Diseases: Clinical and Mechanical Considerations

**DOI:** 10.7150/ijms.128796

**Published:** 2026-03-30

**Authors:** Jiahui Liu, Xiaopeng Guo, Yu Wang, Wenbin Ma

**Affiliations:** Department of Neurosurgery, Peking Union Medical College Hospital, Chinese Academy of Medical Sciences and Peking Union Medical College, Beijing 100730, China.

**Keywords:** KMT5B, SUV420H1, cancer, glioblastoma, neurodevelopmental disorder

## Abstract

KMT5B, also known as SUV4-20H1, is a lysine methyltransferase, catalyzing the generation of H4K20me2 through methylation at the histone H4K20 site, and is increasingly recognized as a pivotal driver of various tumor and non-tumor diseases. Under physiological conditions, KMT5B and its catalytic product, H4K20me2, regulate several essential cellular processes, including DNA replication site selection, G1/S phase transition, DNA double-strand break repair, and stem cell homeostasis in neural and muscle tissues. Furthermore, they support the development of the cytoskeleton, cilia, heart, and lungs. Beyond its catalytic activity, the non-catalytic functions of KMT5B also contribute to genomic stability. Conversely, KMT5B dysregulation is associated with diverse pathologies: deficiency is linked to glioblastoma, sarcoma, and neurodevelopmental disorders, whereas overexpression correlates with hepatocellular carcinoma and chronic myelogenous leukemia. This review summarizes the biological functions and pathological roles of KMT5B identified over the past decades, highlighting its potential as a therapeutic target for both cancer and non-cancer diseases.

## Introduction

First identified in mammals in 2004, KMT5B is a lysine methyltransferase that specifically methylates lysine 20 on histone H4 (H4K20) 1. The H4K20 site presents three methylation states: H4K20me1 catalyzed by KMT5A (PR-Set7), H4K20me2 by KMT5B (SUV4-20H1), and H4K20me3 is primarily synthesized by KMT5C (SUV4-20H2) and partially by KMT5B 1-3. These three methyltransferases dynamically regulate genomic distribution of H4K20, forming a coordinated methylation network.

Histone post-translational modifications (PTMs), including methylation, represent the fundamental mechanism of epigenetic regulation, primarily occurring on lysine and arginine residues of histones H3/H4 4. At the H4K20 locus, KMT5B and its catalytic product, H4K20me2, play key roles in DNA replication, transcriptional regulation, and DNA damage repair 1,3,5-7. Beyond its methylation activity, the non-catalytic functions of KMT5B also maintain genomic stability by mediating DNA detachment and chromatin condensation 8. *KMT5B* overexpression promotes oncogenesis in specific cancers, including head and neck squamous cell carcinoma (HNSCC) 9, and hepatocellular carcinoma (HCC) 10. By contrast, *KMT5B* loss-of-function (LoF) contributes to tumorigenesis and developmental disorders, such as glioblastoma 11,12, sarcoma 13, neurodevelopmental disorders 14-18, and musculoskeletal abnormalities 19,20. A-196 is a drug that selectively inhibits the enzymatic activities of KMT5B and KMT5C 21, while ZINC08398384 and ZINC08439608 are potential compounds targeting KMT5B 22. This review summarizes recent findings regarding the structure and function of KMT5B, elucidates its mechanistic roles in various diseases, and explores its potential as a therapeutic target.

## 1. Structure and Biological Functions of KMT5B

### 1.1 Structure of KMT5B Gene and Its Encoded Protein

**Figure 1:**
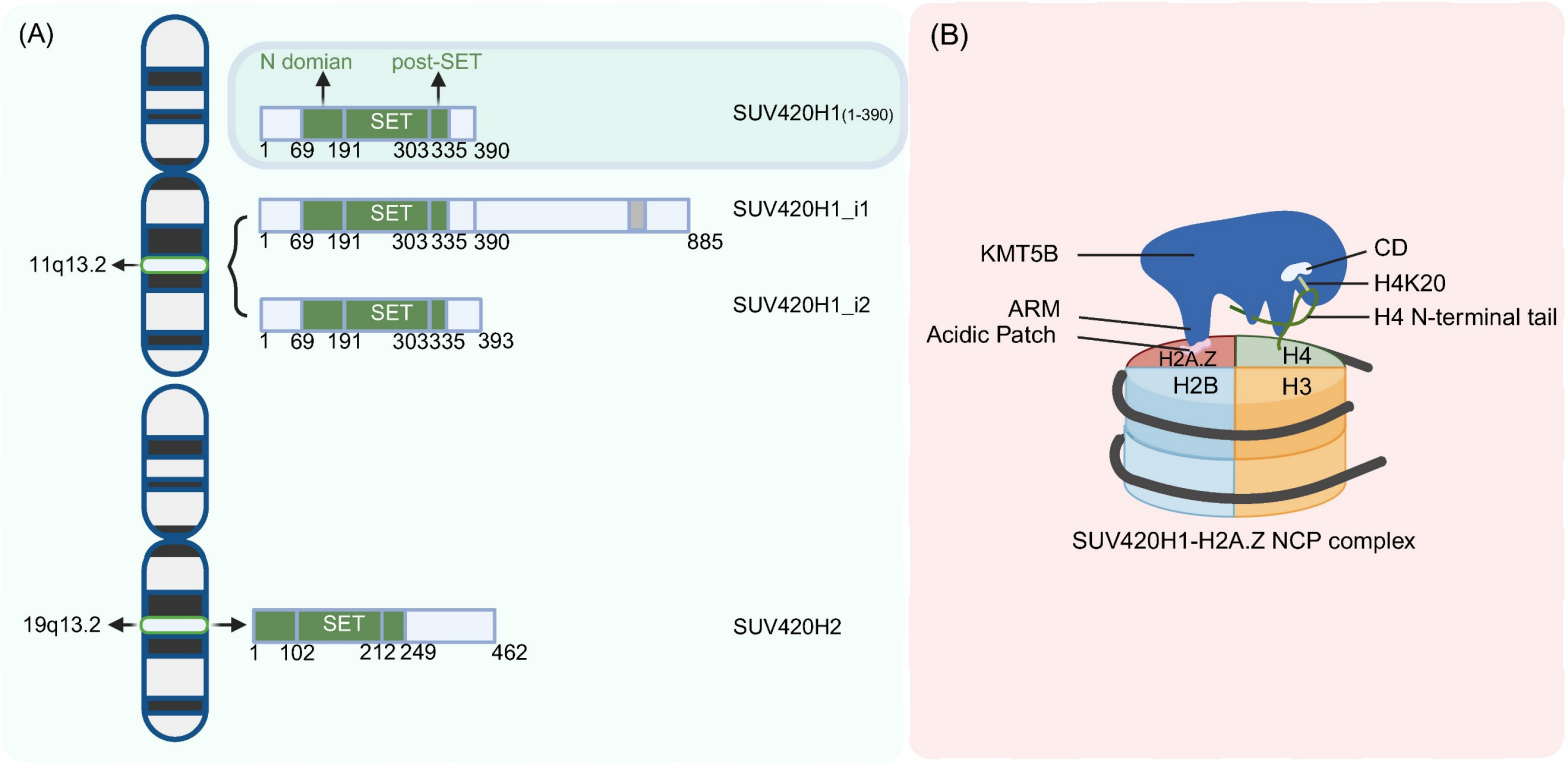
** Structure of KMT5B. A.** Localization and genomic composition of two isoforms of KMT5B and KMT5C on human chromosomes.** B.** Working model of preferential H2A.Z recognition and H4K20 methylation by KMT5B. (Created with BioRender.com)

The human *KMT5B/SUV4-20H1* gene is located on the long arm of chromosome 11, region 1, band 3 (11q13.2), and has 13 exons (Figure 1A) 23,24. Highly homologous to the *KMT5B* gene, the *KMT5C/SUV4-20H2* gene is located on chromosome 19, long arm, region 1, band 3 (19q13.42), with 11 exons 23,24 (Figure 1A).

KMT5B comprises a conserved catalytic domain (CD) and a variable C-terminal regulatory region. The core catalytic domain consists of three components: an N-terminal helical domain (N-domain), a SET (Su(var)3-9, Enhancer-of-zeste, Trithorax) domain, and a Post-SET domain 25 (Figure 1A). Among these, the SET domain serves as the catalytic core, while the Post-SET domain contains a unique CxCxxC zinc-binding motif that is critical for maintaining catalytic function 25. Cryo-electron microscopy analysis revealed that full-length human KMT5B displayed instability and poor homogeneity, and screening of various KMT5B truncations identified residues 1-390 as a stable construct that achieved high expression levels in E. coli while maintaining biological functionality, making it suitable for structural determination 26. Structural variations in the C-terminal regulatory region give rise to two KMT5B isoforms: SUV420H1_i1 retains a full-length C-terminus harboring a PxVxL motif that mediates interactions with heterochromatin protein 1α (HP1α), an interaction enabling targeted localization to pericentromeric heterochromatin where it participates in heterochromatin maintenance, while SUV420H1_i2, characterized by a truncated C-terminus, lacks the PxVxL motif and thus cannot bind HP1α, exhibiting a pan-cellular distribution and potentially regulating cellular differentiation through non-catalytic mechanisms 23.

### 1.2 Biological Functions of KMT5B

KMT5B exerts functions through its catalytic generation of H4K20me2 and H4K20me3, including regulation of DNA replication 3,6,7,27,28, transcriptional repression 13, DNA double-strand break repair (DSB) 29-32, and telomere maintenance 33 and so on. In addition, *KMT5B* inhibition studies also have revealed its non-catalytic activities in maintaining genomic stability 8. This multifunctional nature of KMT5B presents challenges for the development of targeted inhibitory compounds, with current research efforts still ongoing to identify effective therapeutic strategies.

#### 1.2.1 Catalytic activity of KMT5B

**Figure 2:**
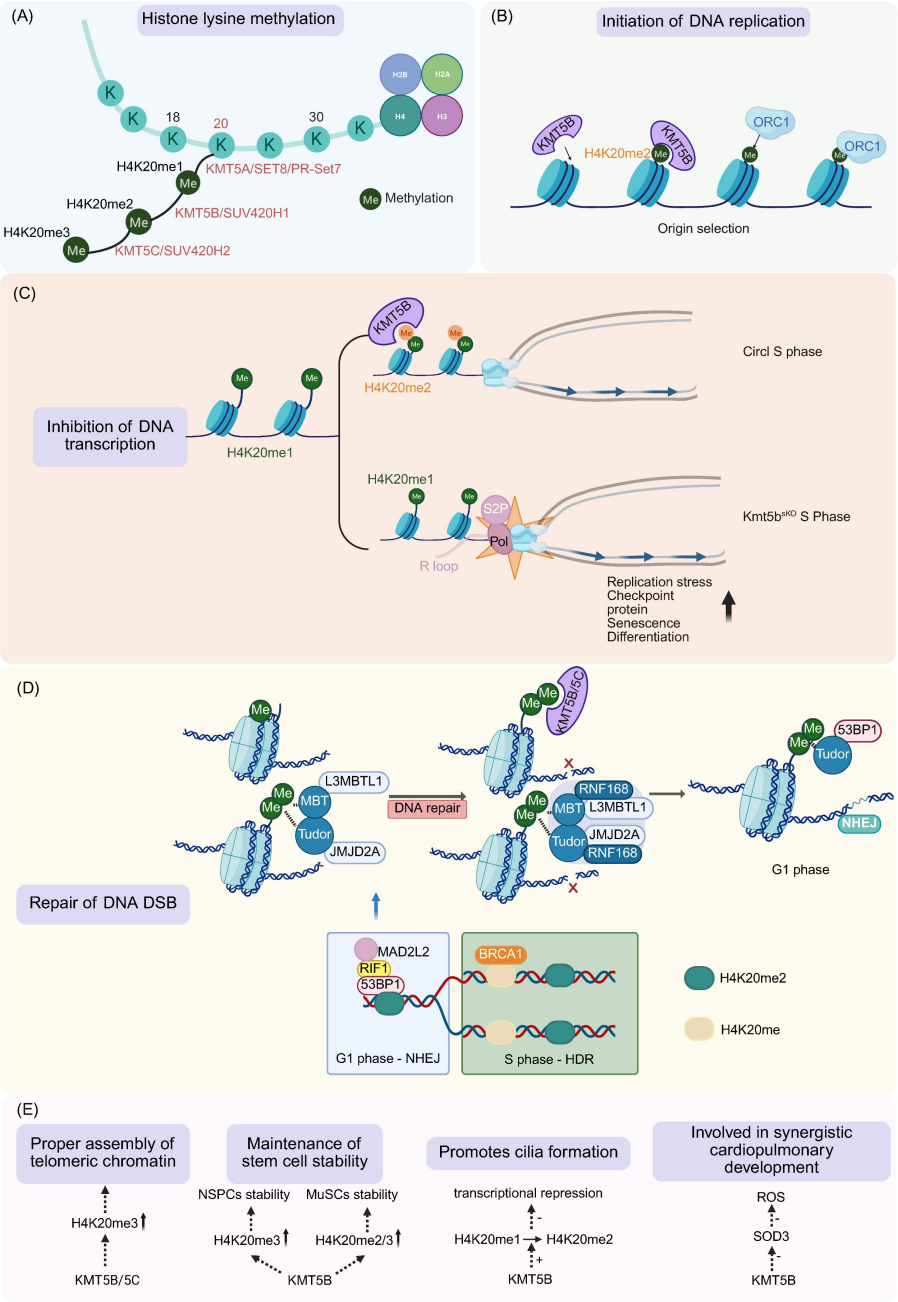
** Function of KMT5B. A.** KMT5B catalyzes dimethylation at the H4K20. Monomethylation is catalyzed by Set8/PR-SET7, while trimethylation is primarily mediated by KMT5C. **B.** During DNA replication, KMT5B assists in the origin selection.** C.** During transcription, KMT5B coordinates transcription and replication by converting H4K20me1 to H4K20me2, thereby restricting the accumulation of H4K20me1 and Pol II S2P on MuSCs genes.** D.** KMT5B regulates H4K20me2 to ensure proper DNA DSB repair, promoting 53BP1-mediated NHEJ in G1 phase and HDR in S phase during DSB repair.** E.** Roles KMT5B plays in ensuring proper telomere assembly, maintaining stem cell stability, promoting cilia growth, and stimulating coordinated cardiopulmonary development. (Created with BioRender.com)

The CD of KMT5B catalyzes H4K20 methylation. Of the five lysine residues on histone H4, K20 is the sole methylation site in mammals and exists in three distinct states: mono-, di-, and tri-methylation 1-3 (Figure 2A). Evolutionary divergence in H4K20 catalysis reveals that while unicellular eukaryotes utilize a single methyltransferase for all states 34, higher vertebrates exhibit functional partitioning through specialized enzyme complexes (KMT5A/B/C) 2,3,34,35 (Figure 2A). Each methylation state exhibits unique genomic localization and functional specialization, with H4K20me1 localizing to euchromatin and regulating cell cycle progression, H4K20me2 demonstrating pan-genomic distribution, and H4K20me3 being enriched at constitutive heterochromatin domains, telomeres, and pericentric regions to mediate transcriptional silencing 1-3.

KMT5B predominantly mediates H4K20me2 and, to a lesser extent, contributes to H4K20me3. In most tissues, including the lung, liver, and epithelial cells of the respiratory and reproductive tracts, KMT5B primarily catalyzes H4K20me2, whereas H4K20me3 is mainly deposited by KMT5C, with only a minor contribution from KMT5B 1,3,36,37. However, in mouse and baboon brain tissues, where KMT5C expression is low, KMT5B serves as the principal enzyme catalyzing both H4K20me2 and H4K20me3 3,36.

To date, the recognition and binding mechanisms by which KMT5B interacts with histones and H4K20me1 are well characterized. KMT5B binds nucleosomes via protein-protein interactions, which recruit the enzyme to specific genomic regions. The catalytic domain recognizes the amino-acid sequence of histone H4 surrounding K20, ensuring site-specific methylation.

*KMT5B's specific recognition and binding of nucleosomal H2A and H2A*.Z. The arginine-rich motif (ARM) of KMT5B can anchor to the nucleosome acidic patch, functioning as a nucleosome tether to ensure the formation of a stable binding interface between KMT5B and the nucleosome; its catalytic domain (CD) primarily interacts with the lasso-shaped structure at the N-terminus of histone H4 and nucleosomal DNA, providing a structural basis for the methylation catalysis at the H4K20 site 8,38 (Figure 1B). H2A.Z is a highly conserved variant in the core histone H2A family, accounting for approximately 5%-10% of total H2A in human cells, with only 64% sequence similarity to canonical histone H2A 27,38. It possesses a unique amino acid sequence that enables specific interactions with proteins such as KMT5B, thereby regulating critical biological processes including chromatin structure, DNA replication, and DNA repair 8,27,38 (Figure 1B). Functional experiments established that H2A.Z regulates KMT5B activity. Compared with H2A, H2A.Z significantly increases the efficiency of KMT5B-mediated H4K20me2 deposition 27. Residues D97 and S98 of H2A.Z are critical for recognition by KMT5B, and mutations at these residues markedly reduce the enzyme's catalytic activity 27. Cryo-electron microscopy (cryo-EM) analyses at resolutions of 3.2-3.68 Å further revealed the key binding interfaces underlying the substrate preference of KMT5B for H2A.Z-nucleosomes: in addition to the interaction between the acidic patch (composed of specific acidic amino acids of H2A/H2B) and the ARM of KMT5B, the KR loop of KMT5B (key residues K219/R220) and the D97/S98 sites of H2A.Z also constitute core interaction regions 26,38 (Figure 1B). Microscale thermophoresis (MST) experiments demonstrated that the binding affinity of KMT5B for H2A.Z-nucleosomes (KD = 130 nM) is higher than that for H2A-nucleosomes (KD = 227 nM) 26, endpoint histone methyltransferase (HMT) assays further confirmed the enhanced catalytic activity of KMT5B toward H2A.Z-nucleosomes 8,26. Kinetic analyses showed that the catalytic constant (kcat) of KMT5B is higher when using H2A.Z-nucleosomes as substrates (252.9 ± 1.0), while the Michaelis constant (Km) is comparable to that with H2A-nucleosomes, indicating that H2A.Z enhances the catalytic efficiency of KMT5B through an allosteric effect 8. Furthermore, cryo-EM results confirmed that S-adenosylmethionine (SAM) is an essential methyl donor for KMT5B 8,26,27,38. Its binding site is located near the catalytic center, and the binding of SAM to KMT5B is closely associated with the conformational stability of the catalytic center 8,26,38.

*KMT5B's specific recognition and binding of substrate H4K20me1. In vitro*, while KMT5B can directly methylate unmodified H4K20 in every state, it exhibits strong substrate preference for H4K20me1 1. *In vitro*, a study employed SPOT peptide array technology to for the first time define the sequence-specific motif of KMT5B as (R/Y)-Kme1-(I/V/L/M)-(L/F/I)-X-D 25. This motif exhibits strict selectivity for the -1 position (arginine, R/tyrosine, Y) and the +4 position (aspartic acid, D), and exclusively recognizes monomethylated lysine (Kme1) 25. Studies applying Cryo-EM on the binding interface between KMT5B and H4K20me1 have further clarified their interaction mechanism: the N-terminus of histone H4 forms a “lasso-shaped structure” that accurately delivers H4K20me1 into the hydrophobic pocket of KMT5B's CD, while the binding of S-adenosylmethionine (SAM) can stabilize the conformation of the catalytic center to enhance this recognition process 8,26,38.

#### 1.2.2 Non-catalytic activity of KMT5B

The C-Terminal Region of KMT5B mediates nucleosomal DNA detachment and chromatin condensation 8. In recent years, epigenetics research has focused on the functionally independent catalytic and non-catalytic activities of histone-modifying proteins/complexes 39-41. Mouse embryonic fibroblasts (MEFs) with double knockout of KMT5B and KMT5C are highly sensitive to DNA damage, whereas cells treated only with catalytic inhibitors targeting these two proteins exhibit only mild sensitivity 3,21. This observation suggests that these proteins possess uncharacterized functions that are independent of their catalytic activity. Such non-catalytic functions may be associated with the diverse roles of KMT5B in different diseases, as well as the current lack of drugs capable of fully inhibiting its functions.

Cryo-EM analyses revealed that KMT5B binding to nucleosomes induces the detachment of terminal nucleosomal DNA from the histone octamer 8. This DNA detachment activity is mediated by the C-terminal region of KMT5B, which interacts with the αN helix of histone H3 and is independent of its histone methyltransferase activity 8. Notably, this activity exposes internal histone surfaces within the nucleosome, such as the PxVxL motif located in the α1 helix of histone H2B, thereby creating conditions for the binding of macromolecular complexes (e.g., HP1) to chromatin 8. Single-molecule optical tweezers showed KMT5B alters nucleosomal DNA's outer wrap and mediates inter-nucleosomal interactions, causing low-force regime transitions 8. *In vitro* assays revealed KMT5B alone or with HP1α promotes chromatin condensation into aggregates 8. These non-catalytic actions regulate chromatin dynamics and DNA accessibility, supporting genomic stability.

#### 1.2.3 Initiation of DNA replication

KMT5B plays a critical role in DNA replication initiation by catalyzing H4K20me2 formation (Figure 2B) 3,6,7,27,28. DNA replication begins in G1 cell cycle phase, where the origin recognition complex (ORC) recruits minichromosome maintenance (MCM) helicase to form the pre-replication complex (pre-RC), followed by S-phase activation of licensed origins through sequence features and epigenetic regulation like H4K20me2 42,43.

Emerging evidence from multiple model systems, including zebrafish embryos, human cell lines, mouse embryonic fibroblasts (MEFs), chronic myeloid leukemia K562 cells, and head and neck squamous carcinoma models, has revealed that KMT5B coordinates replication initiation by catalyzing H4K20me2 deposition at origins of replication (ORIs) to facilitate ORC complex recruitment and suppressing p21WAF1/CIP1 expression to alleviate G1 checkpoint inhibition 3,7,28.

Structural analyses show that ORC1's bromo-adjacent homology (BAH) domain directly recognizes H4K20me2-modified chromatin, and this interaction is evolutionarily conserved from zebrafish to humans 6. Disruption of the BAH-H4K20me2 interface significantly impairs ORC1 occupancy at replication origins, compromises ORC chromatin loading, and ultimately arrests cell cycle progression at the G1/S transition 6. Genetic evidence supporting this pathway includes *Kmt5b/5c* double-knockout MEFs that display reduced S-phase populations, G1-phase accumulation, and developmental delay 3, and KMT5B depletion in K562 and HNSCC cells that induces G1 arrest via p21 upregulation 7,28.

The H2A.Z-KMT5B-H4K20me2-ORC1 axis facilitates replication initiation site selection via multiple mechanisms: H2A.Z-containing nucleosomes directly recruit KMT5B to deposit H4K20me2 modifications, which in turn enhance ORC1 binding 27. Genome-wide analyses reveal strong colocalization between H2A.Z, H4K20me2, ORC1, and active replication origins, and H2A.Z-regulated origins display higher replication signals and earlier replication timing (Figure 2B) 27. This mechanism is functionally conserved across species, as conditional H2A.Z knockdown in mouse T cells similarly impairs proliferation and reduces replication signals 27.

#### 1.2.4 Inhibition of DNA transcription

KMT5B plays a critical role in maintaining genomic stability by orchestrating the dynamic balance between DNA replication and transcription 13. Aberrant transcription activation causes local replication fork stalling and elevated replication stress, ultimately resulting in genomic instability, a hallmark of cancer 44. The coordination of transcription and replication in skeletal muscle stem cells (MuSCs) is orchestrated by KMT5B through the catalysis of H4K20me1 to H4K20me2; this modification restrains the aberrant accumulation of H4K20me1 and Ser2-phosphorylated RNA polymerase II (Pol II S2P) across MuSC gene bodies 13. Such molecular regulation is essential to suppress S-phase transcriptional activity and mitigate the formation of transcription-replication collisions (TRCs) and R-loops within MuSCs 13 (Figure 2C). The depletion of KMT5B serves as the primary initiating event for a cascade of pathological responses, culminating in the activation of the ATR-RPA32-p53-dependent protective checkpoint. A tumor-suppressive effect is subsequently exerted by this signaling axis through the arrest of MuSC proliferation and the induction of cellular senescence 13. Such senescence functions as a transient barrier rather than a definitive obstruction to malignancy. The abrogation of this senescence-associated growth arrest occurs upon p53 inactivation, thereby permitting KMT5B-deficient MuSCs to bypass cell cycle checkpoints, proliferate uncontrollably, and accumulate extensive genomic aberrations 13. Ultimately, these combined abnormalities create the necessary conditions for MuSCs to undergo malignant transformation 13 (Figure 2C).

#### 1.2.5 Repair of DNA double-strand breaks

KMT5B governs DNA DSB repair by dynamically regulating H4K20me2-mediated chromatin signaling, thereby coordinating pathway choice and effector recruitment 30-32,45. The repair of DSBs is primarily mediated through two distinct pathways: non-homologous end-joining (NHEJ) and homologous recombination (HR), which are preferentially active in G1 and S/G2 phases of the cell cycle, respectively 30. This cell cycle-dependent pathway choice is regulated by the opposing actions of 53BP1, which promotes NHEJ by inhibiting DNA end resection, and BRCA1, which facilitates HR 30. Cell cycle-dependent fluctuations in H4K20me2 levels dynamically control the recruitment and stabilization of the 53BP1-RIF1-MAD2L2 complex at DSB sites 30. During the G1 phase, high H4K20me2 levels maintain 53BP1 complex retention to ensure efficient NHEJ, while S-phase progression leads to H4K20me2 dilution via chromatin replication, displacing the 53BP1 complex and subsequently recruiting BRCA1 to enable HR 30 (Figure 2D). Emerging evidence indicates that while KMT5B/5C primarily establishes the genome-wide H4K20me2 landscape, the methyltransferase MMSET is recruited to DSBs via the γH2AX-MDC1 pathway to locally increase H4K20me2 deposition, thereby facilitating 53BP1 accumulation at break sites 31,32 (Figure 2D).

The recognition of H4K20me2 by 53BP1 involves a sophisticated displacement mechanism: in undamaged chromatin, H4K20me2 is sequestered by L3MBTL1 and JMJD2A through their MBT and Tudor domains, respectively 31 (Figure 2D). Following DSB formation, KMT5B/5C-mediated conversion of H4K20me1 to H4K20me2, coupled with RNF168-dependent polyubiquitination and VCP-mediated proteasomal degradation of L3MBTL1/JMJD2A, exposes H4K20me2 for specific recognition by 53BP1's tandem Tudor domains, ultimately facilitating its stable recruitment to DNA lesions 31 (Figure 2D).

#### 1.2.6 Proper assembly of telomeric chromatin

H4K20me3 is a hallmark heterochromatic modification at mammalian telomeres 46. Loss of H4K20me3 compromises telomere stability, which triggers genomic instability and promotes cancer development and progression 46,47. In MEFs lacking only KMT5C, H4K20me3 levels at subtelomeric regions of chromosomes 1 and 2 were markedly reduced 47. By contrast, knockout of KMT5B alone did not alter H4K20me3 levels 47. However, simultaneous depletion of both KMT5B and KMT5C further exacerbated the loss of H4K20me3 at subtelomeric regions of these two chromosomes 47. These findings demonstrate that KMT5C is the primary catalytic enzyme responsible for subtelomeric H4K20 trimethylation, whereas KMT5B acts to reinforce this activity.

#### 1.2.7 Maintenance of stem cell stability

*Maintenance of neural stem/progenitor cells (NSPCs)*: The subventricular zone (SVZ) contains NSPCs that generate neurons and can differentiate into neurons, astrocytes, and oligodendrocytes. Glioblastoma (GBM) contains NSPCs, and among the many regulatory mechanisms, histone methylation, which encompasses the role of KMT5B, is a crucial regulator of cell fate 36.

Baboons were used to obtain endogenous NSPCs to assess the role of H4K20me3 in NSPCs *in vivo*, human GBMs were employed for pathobiological analyses, and mouse models were used for genetic manipulation 36. Studies of NSPCs isolated from the SVZ of the adult baboon brain and mice revealed that KMT5B/5C-H4K20me3 might be a protective mechanism against the generation of aberrant cell cycles in NSPCs by decreasing aberrant gene expression and regulating cell proliferation 36. In mice, KMT5B is prevalent in adult tissues, including the brain, whereas KMT5C is not expressed in the brain, and there are no apparent phenotypic alterations in *Kmt5c* knockout mice 3. So, it is believed that KMT5B mainly produces H4K20me3 in the mouse brain 3. Overall, the interaction between KMT5B/H4K20me3 and EZH2/H3K27me3 would be a protective mechanism critical for characterizing adult NSPCs in the SVZ by preventing premature cell lineage stereotyping and cell cycle progression 36 (Figure 2E).

*Maintenance of skeletal muscle stem cells (MuSCs*): MuSCs are indispensable for the regeneration of adult skeletal muscle following injury, and this regenerative process requires the activation of MuSCs that are predominantly in a quiescent state 19. KMT5B maintains the quiescence of MuSCs by promoting the formation of facultative heterochromatin (fHC) and repressing the expression of the MyoD gene—a key transcription factor gene associated with myogenic differentiation 19 (Figure 2E). This ensures the stability of the stem cell pool and the long-term regenerative capacity of skeletal muscle. In MuSCs from a mouse model of Duchenne Muscular Dystrophy (DMD), the expression of KMT5B is downregulated, the formation of fHC is reduced, and the expression of MyoD is increased, which recapitulates the phenotypic characteristics observed in KMT5B-deficient MuSCs 19.

#### 1.2.8 Promotes cilia formation

KMT5B has been reported to promote ciliogenesis in larval epidermal multiciliated cells (MCCs) of Xenopus laevis 48. Targeted knockdown of *KMT5B/5C* via morpholino oligonucleotides (MOs) in Xenopus revealed their essential role in multiciliogenesis 48. Double-morphant tadpoles exhibited impaired motility as a result of disrupted ciliary beating, accompanied by axonemal defects in epidermal MCCs 48. Organoid assay demonstrated specific downregulation of ciliary/cytoskeletal genes upon *KMT5B/5C* depletion 48. KMT5B-mediated conversion of H4K20me1 to H4K20me2 on postmitotic chromatin alleviates transcriptional repression, confirming its enzymatic requirement for axoneme assembly 48 (Figure 2E).

#### 1.2.9 Synergistic cardiopulmonary development

KMT5B is critical for cardiopulmonary development. *Kmt5b* deletion in murine cardiopulmonary progenitors reduces H4K20me2/3 levels, triggering COPD (chronic obstructive pulmonary disease)-like PH (pulmonary hypertension) phenotypes, including lymphoid infiltration, vascular remodeling, and alveolar defects that progress to fatal heart failure 49. KMT5B represses expression of secreted superoxide dismutase 3 (Sod3) to restrict hydrogen peroxide production; its loss increases oxidative stress and disrupts pulmonary and vascular development 49 (Figure 2E).

## 2. KMT5B Alterations and Pathogenesis

### 2.1 Carcinogenic Effects

**Figure 3:**
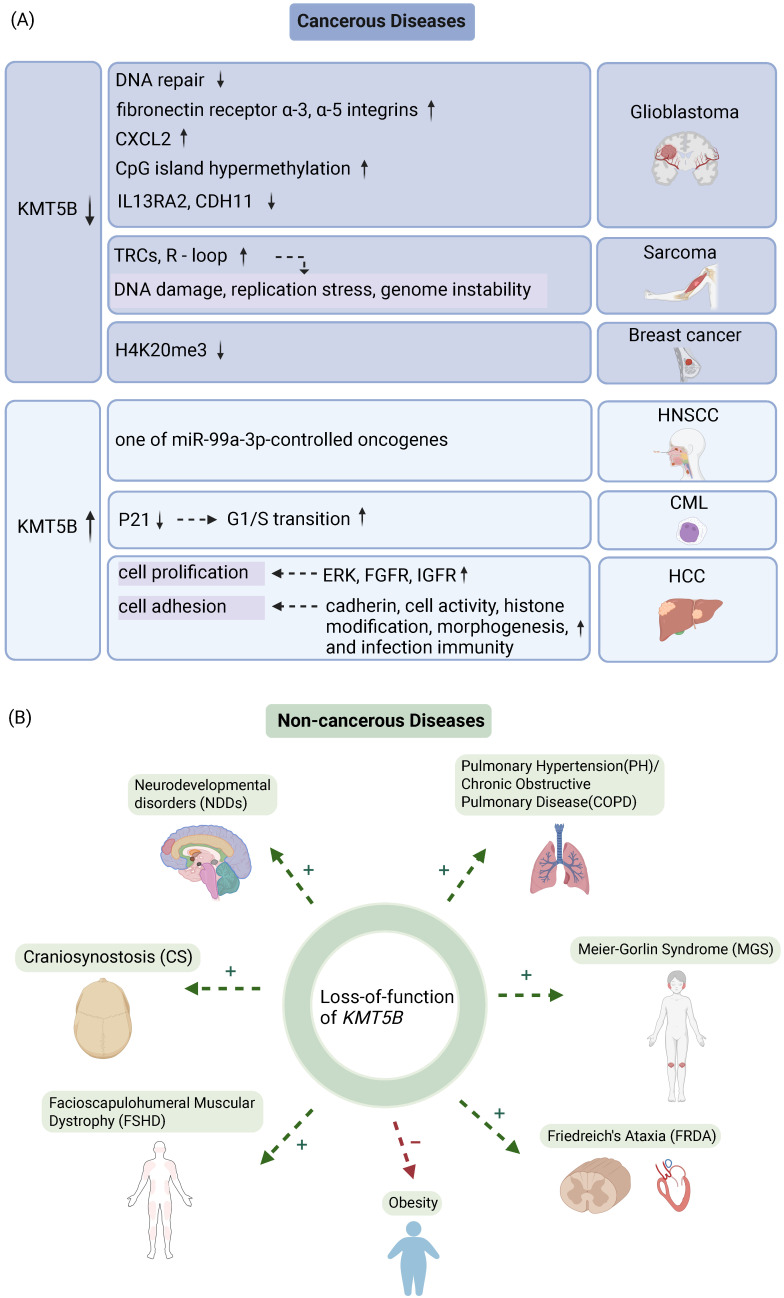
** The role of KMT5B in cancerous and non-cancerous diseases. A.** The role of *KMT5B* loss-of-function and overexpression in the progression of various tumor types.** B.** Functional consequences of *KMT5B* depletion across non-malignant disorders. “+” denotes disease-aggravating effects; “-” denotes protective effects. (Created with BioRender.com)

Pan-cancer analyses delineate distinct *KMT5B* alteration landscapes, characterized by predominant copy number variations and recurrent missense mutations (COSMIC/cBioPortal) 50. This epigenetic regulator shows frequent genomic aberrations in gastrointestinal malignancies (esophageal adenocarcinoma: 18%; gastric cancer: 12%), thoracic tumors (11%), and genitourinary tumors, with R320C/G534V hotspot mutations demonstrating lineage-dependent oncogenic proclivities 50. In GBM, *KMT5B* deletion promotes tumor aggressiveness through coordinated mechanisms, including impaired DNA repair, extracellular matrix remodeling, and epigenetic dysregulation (DNA hypermethylation/hypo-hydroxymethylation) 11,12,36 (Figure 3A). Similarly, sarcomas exhibit *KMT5B* loss-driven tumorigenesis mediated mainly by the replication stress elevation 13 (Figure 3A). Conversely, this does not hold true in leukemia 7 and head and neck squamous carcinomas 28, where *KMT5B* deletion suppresses tumor cell proliferation (Figure 3A). These paradoxical findings underscore the context-dependent role of KMT5B in cancer progression, likely shaped by tissue-specific molecular profiles and cellular dependencies.

#### 2.1.1 Impairment of DNA DSB repair

KMT5B deficiency impairs DNA DSB repair and enhances tumor invasion and migration 11. In a study dissecting functional differences between pediatric GBM subclonal populations and diffuse endogenous pontine glioma (DIPG) cells, investigators utilized NS-F10, a single cell-derived neurosphere colony with *KMT5B* private mutation that introduced a premature termination codon at amino acid 187 (R187*) and reduced H4K20me2 11. Drug screening against 80 targeted agents showed *KMT5B*-mutated NS-F10 exhibited heightened sensitivity to multiple PARP inhibitors compared to the wild-type NS-F8 and HSJD-DIPG-007 cells 11. The loss of KMT5B abrogates DNA repair process by decreasing H4K20me2 and recruitment of 53BP1 11 (Figure 3A).

#### 2.1.2 Extracellular matrix remodeling

KMT5B deletion upregulates genes associated with extracellular matrix (ECM) remodeling and enhances tumor invasion and migration 11 (Figure 3A). RNA-sequencing analysis of subclones revealed markedly elevated expression of ECM remodeling genes in KMT5B-mutant NS-F10 cells, and *in vitro* assays demonstrated increased matrix gel invasion and fibronectin-dependent migration in NS-F10 models 11. Immunofluorescence and immunohistochemistry confirmed differential expression of the fibronectin receptors α3-integrin and α5-integrin 11 (Figure 3A). *In vivo*, orthotopic implantation into mouse brainstem showed that NS-F10 subclones formed diffusely infiltrative tumors more rapidly than wild-type NS-F8 controls 11.

#### 2.1.3 Abnormal epigenetic characterization

KMT5B deficiency promotes GBM aggression, while KMT5B overexpression suppresses the growth of GBM cells by inducing cell cycle arrest and regulating the expression of IL13RA2 and CDH11.

KMT5B overexpression induces G2 cell cycle arrest, reduces cell proliferation, viability, and clonogenicity *in vitro*, and inhibits tumor growth in mouse models. *KMT5B* deletion drives DNA methylation at CpG islands, a well-established epigenetic hallmark of cancer 12 (Figure 3A). GBMs exhibit concurrently DNA hypermethylation and hypohydroxymethylation, which correlates with KMT5B downregulation and global loss of H4K20me2 12.

KMT5B overexpression attenuates IL13RA2 expression. While IL13 induces apoptosis across multiple cell types, including GBM cells, its high-affinity receptor IL13RA2 exerts oncogenic activity by both blocking IL13-mediated apoptosis and actively promoting tumor growth 12. Overexpression of IL13RA2 is correlated with higher glioma grade and shortened patient outcomes, whereas KMT5B overexpression drives H4K20me2 deposition at the IL13RA2 promoter, resulting in reduced IL13RA2 mRNA levels 12 (Figure 3A).

KMT5B overexpression upregulates CDH11. CDH11 has been reported to inhibit GBM cell invasion *in vitro*. H4K20me2 acts as a transcriptional activator of CDH11, cooperating with other histone modifications and epigenetic readers 12 (Figure 3A).

#### 2.1.4 ATR-P53 pathway activation in tumors

KMT5B deficiency activates the ATR-P53 pathway to promote rhabdomyosarcoma development 13. *KMT5B* knockdown in MuSCs elevates H4K20me1 levels, disrupts S-phase transcriptional repression, and generates oncogenic R-loops at TRCs 13 (Figure 3A). This triggers replication stress and mitotic defects that elicit ATR-RPA32-p53-mediated senescence, thereby driving rhabdomyosarcoma formation 13. Pharmacological inhibition of S-phase transcription in KMT5B-deficient cells exacerbates TRC/R-loop accumulation, confirming its role in suppressing replication-transcription conflicts 13 (Figure 3A).

#### 2.1.5 ERK signaling pathway in tumors

KMT5B overexpression has been proposed to enhance extracellular signal-regulated kinase (ERK) phosphorylation and transcriptional activation of the ERK signaling pathway to promote carcinogenesis 9 (Figure 3A). A study reported that KMT5B trimethylates ERK1 at lysine residues 302 and 361, and methylation-site substitution reduces ERK1 phosphorylation 9 (Figure 3A). However, a subsequent study has shown that the sequences surrounding these two lysine residues do not conform to KMT5B's specific recognition sequence, and KMT5B-mediated methylation of ERK1 was not detected 25.

#### 2.1.6 Promotion of G1/S transition in tumors

KMT5B represses CDKN1A expression through H4K20 methylation, thereby facilitating G1/S transition and tumor progression 7 (Figure 3A). KMT5B knockdown in K562 leukemia cells significantly reduces H4K20me2/3 enrichment at the CDKN1A promoter and correspondingly increases CDKN1A expression, leading to G1 phase accumulation by inhibiting cyclin-CDK complexes and blocking RB phosphorylation-mediated E2F release 7. This tumor-promoting function of KMT5B is conserved across cancer types, as illustrated in head and neck squamous carcinoma, where KMT5B depletion similarly reduces S-phase cells and increases G1-phase populations, ultimately suppressing cancer cell growth 9 (Figure 3A).

### 2.2 Obstruction of Nervous System Development

*KMT5B* has been identified as one of the top-ranked autism risk genes through large-scale whole-genome sequencing studies of autism spectrum disorder (ASD) patients 14,51,52. *KMT5B* mutations identified in ASD patients result in LoF, and thus contribute to social deficits through glutamatergic synaptic defects in the prefrontal cortex 15.

**Figure 4:**
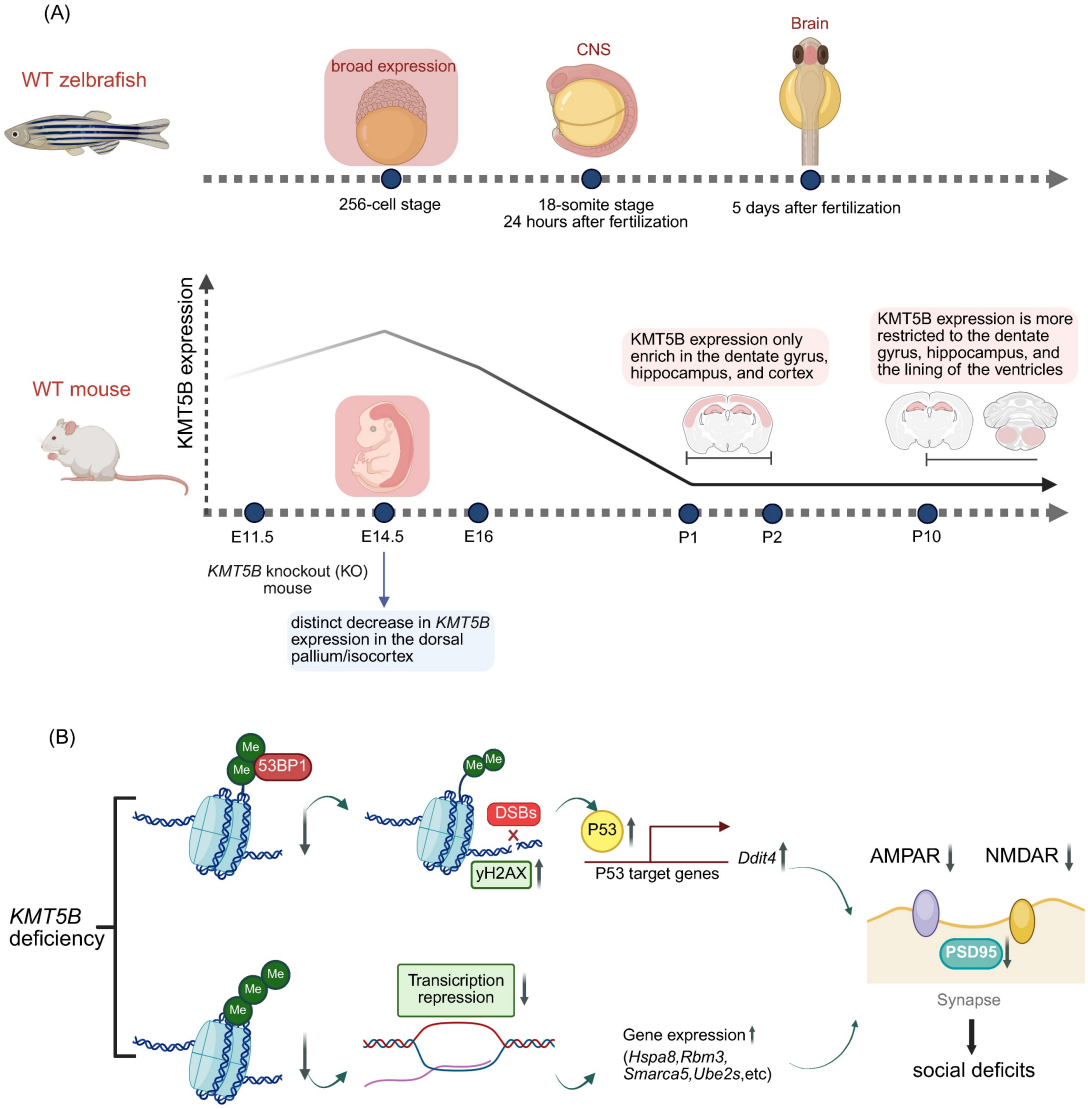
** The role of KMT5B in the neurodevelopmental diseases. A.** Spatiotemporal expression patterns of KMT5B during development in zebrafish and mouse models.** B.** *KMT5B* deficiency-induced social impairment mechanisms. (Created with BioRender.com)

KMT5B is highly expressed in the prefrontal cortex (PFC) 53,54, a region critical for executive function and social cognition 55 and is frequently implicated in ASD 56. Synaptic dysfunction in PFC pyramidal neurons is a well-established hallmark in both ASD patients and mouse models 15. Adolescent mice (3-7 weeks) were used, as this represents a critical period for PFC circuit maturation and the development of social and executive functions 15 (Figure 4A). Behavioral analysis revealed that *Kmt5b*-deficient mice exhibited significant reductions in social interaction and preference in a three-chamber assay 15. Potential mechanisms underlying KMT5B's role in ASD pathogenesis include the following:

#### 2.2.1 Decreased expression of AMPAR and NMDAR subunits

Both AMPARs and NMDARs are ionotropic glutamate receptors. Glutamate binding activates NMDARs to open voltage-dependent ion channels, while AMPAR-mediated depolarization expels magnesium ions from the NMDA pore, enabling Na⁺/Ca²⁺ influx and K⁺ efflux 15. Ca²⁺-driven CaMKII activation is critical for LTP induction 15 (Figure 4B). In *Kmt5b*-deficient PFC neurons, AMPAR/NMDAR-mediated EPSC amplitude is reduced, accompanied by decreased levels of GluR2, NR2A, and NR2B subunits, which underlie PFC functional deficits 15 (Figure 4B).

#### 2.2.2 Upregulation of P53 expression

*Kmt5b* deficiency induces DSBs and activates p53 signaling, upregulating its downstream target *Ddit4* (Redd1) 15. Previous work has shown KMT5B catalyzes H4K20me2, recruiting 53BP1 to DSBs via direct 53BP1-H4K20me2 complex formation to promote NHEJ repair 31. In *KMT5B*-deficient cells, elevated γH2AX levels indicate increased DSBs, coinciding with p53 activation 15 (Figure 4B). p53 transcriptionally regulates downstream effectors including Ddit4, which is upregulated following *KMT5B* deletion and correlates with prefrontal cortical synaptic loss in both depressed humans and chronically stressed mice 15 (Figure 4B). In *Kmt5b*-deficient PFC neurons, elevated Ddit4 may underlie impaired synaptic transmission and reduced levels of glutamatergic receptors and PSD-95 15 (Figure 4B).

#### 2.2.3 Upregulation of differentially expressed genes (DEGs)

Among the differentially expressed genes (DEGs) in *Kmt5b*-knockdown prefrontal cortex (PFC), the majority were significantly upregulated, while only 16 were markedly downregulated 15. Among the 16 downregulated genes, the top four highly enriched biological process terms included protein folding involving heat-shock proteins (Hsps), translation regulators (Eif4a1, Eif2s3x, Eef1g), covalent chromatin modification (Smarca5), and ubiquitin-dependent catabolic processes (Ube2s, Psma3, Psma5) 15. These dysregulated genes may underlie the impaired synaptic function and protein homeostasis in *Kmt5b*-deficient PFC 15 (Figure 4B).

#### 2.2.4 Impairment of dendrite development, NPC proliferation, and acceleration of neuronal migration

An early large-cohort study of *KMT5B*-related NDDs demonstrated that *KMT5B* LoF and missense variants disrupt prenatal neurogenesis and neuronal development 52. *In vitro*, *Kmt5b* knockdown in mouse cortical neurons reduced dendritic complexity and increased spine density—defects unrescuable by pathogenic mutants 52. In utero *Kmt5b* silencing in mouse embryos impaired neurogenesis: NPC proliferation decreased, neuronal migration accelerated, and cortical layering disorganized 52. Transcriptomic analysis linked these deficits to dysregulated axon guidance and chromatin modification pathways, confirming KMT5B's essential role in orchestrating cortical development 52.

#### 2.2.5 Dysregulated apoptosis of post-mitotic neurons

KMT5B and RB1 (retinoblastoma protein) form the critical “RB1-KMT5B-Bcl2a/caspase” regulatory axis, pivotal for neuronal survival and linked to neurological disorders 57. RB1, a cell cycle regulator, directly binds KMT5B to enhance its enzymatic function, thereby inhibiting post-mitotic neuronal apoptosis via the Bcl2a/caspase pathway—a key pathway for neuronal homeostasis 57. *RB1* mutations (2%-3% in neurodegenerative disease patients, e.g., R621S, L819V) disrupt this binding, abolishing anti-apoptotic activity 57. Zebrafish models confirm *rb1* deletion induces neuronal apoptosis, microglial infiltration, and motor deficits; wild-type KMT5B overexpression partially rescues these, while *kmt5b* knockdown mimics *Rb1* loss 57. RB1 also recruits KMT5B for chromatin modification and epigenetic repression 57. Dysregulation of this interaction contributes to both neurodegenerative and neurodevelopmental disorders.

### 2.3 Cilium Defect

H4K20me1-to-H4K20me2 conversion catalyzed by KMT5B is essential for multiciliogenesis 48. KMT5B/C activity is critical for axoneme assembly in MCCs, as its depletion disrupts axonemal structure and downregulates ciliary/cytoskeletal genes via H4K20me1 accumulation 48. Wild-type cells relieve this repression through KMT5B-mediated H4K20me2 deposition 48. PHF8 (an H3K4me3 demethylase lacking a catalytic C-terminal domain) deletion partially rescues ciliogenesis defects in *KMT5B* mutants, implicating H4K20me1-reader proteins in MCC gene silencing 48.

### 2.4 Cardiopulmonary Progenitor Cell Development Disorder

Deletion of *KMT5B* in cardiopulmonary progenitor cells leads to a COPD-like/PH phenotype in mice, including the formation of perivascular tertiary lymphoid tissue, hyperproliferation of smooth muscle cells/myofibroblasts, impaired alveolization, and defects in maturation of the microvascular system, which results in massive right ventricular dilatation and premature death 49. Epigenetic regulation of Sod3 is disrupted by KMT5B loss, leading to ROS imbalance and subsequent cardiopulmonary developmental defects that may contribute to the pathogenesis of COPD and PH 49.

### 2.5 Skeletal and Muscular Development Disorders

Deleting *KMT5B* reduces fHC synthesis and induces MyoD transcriptional activation and relocalization away from the heterochromatic nuclear periphery, promoting troponin activation and leading to stem cell failure and impaired muscle regeneration 19. Additionally, in normal skeletal muscle, *Eid3* (EP300 interacting inhibitor of differentiation 3, a novel myogenic inhibitor) is silenced by KMT5B via H4K20me3 modification, thereby ensuring the normal progression of myogenic differentiation 58. In Facioscapulohumeral Muscular Dystrophy (FSHD), however, deletion of the D4Z4 macrosatellite repeat results in the overexpression of *FRG1* (FSHD Region Gene 1) 58. This overexpressed FRG1 binds directly to KMT5B and interferes with its sub-nuclear localization and enzymatic function 58. Loss of KMT5B-mediated repressive control of Eid3 leads to its upregulation, which inhibits myogenic differentiation and triggers muscular dystrophy 58.

## 3. KMT5B in Cancers

### 3.1 GBM

GBM, the most aggressive primary adult brain tumor 59, harbors *KMT5B* LoF alterations that correlate with poor clinical outcomes.

In pediatric and diffuse midline gliomas, rare *KMT5B*-deficient subclones (< 1%) exert paracrine effects that drive tumor progression 11. Through modulation of chemokine signaling and integrins, these subclones induce DNA repair defects and enhance invasive migration in neighboring tumor cells 11 (Figure 3A).

Under the current 2021 WHO classification of central nervous system tumors, *KMT5B* alterations remain excluded as diagnostic or prognostic biomarkers owing to a lack of sufficient clinical evidence 60. However, several studies have suggested that *KMT5B* mutations are associated with patient prognosis. A characterization and predictive analysis of 20 adult patients with pediatric-type diffuse gliomas identified *KMT5B* mutations in six individuals, five of whom harbored the recurrent p.Glu833_Asp835delinsSerProSer variant—an in-frame deletion-insertion localized within the KMT5B functional domain that is suggestive of a LoF effect 61. Furthermore, multivariate regression analysis revealed a significant correlation between *KMT5B* LoF and abbreviated overall survival (OS) in pediatric-type gliomas 61. An additional study focusing on elderly GBM patients demonstrated that *KMT5B* LoF alterations are associated with a trend toward diminished outcomes 62. Real-world analysis of IDH wild-type gliomas recognized *KMT5B* LoF as an independent poor prognostic factor, along with older age, male sex, and deep brain structure involvement 63.

### 3.2 Sarcoma

Sarcomas represent a subset of rare malignant neoplasms (< 1% of all cancers) that demonstrate persistently poor clinical outcomes despite multimodal therapy 64. Although KMT5B is highly expressed in multiple malignancies, TCGA analysis linked KMT5B downregulation to TP53/KMT5B co-alterations across human sarcoma subtypes, supporting a pan-sarcoma tumor suppressor function 13 (Figure 3A). KMT5B prevents sarcoma genesis through S-phase transcriptional repression, and its low expression associates with inferior disease-free survival 13 (Figure 3A).

### 3.3 Head and Neck Squamous Cell Carcinoma

HNSCC ranks as the seventh most prevalent malignancy globally, with increases in both incidence and mortality 65. KMT5B is highly expressed in HNSCC patients and associated with a dismal prognosis (Figure 3A).

Recent RNA sequencing studies identified miR-99a-5p and miR-99a-3p as tumor-suppressive miRNAs in HNSCC, with both strands demonstrating significant attenuation of malignant phenotypes when overexpressed 28 (Figure 3A). Integrative multi-omics analysis revealed miR-99a-3p's pan-oncogenic regulatory network encompassing 32 targets (including KMT5B), among which ten showed clinical prognostic value, with multivariate Cox regression establishing KMT5B overexpression as a robust independent predictor of worse survival in HNSCC cohorts 28 (Figure 3A). The oncogenic effects of KMT5B were found to be mediated via the enhancement of ERK1 phosphorylation at Thr202/Tyr204 and the upregulation of ERK1 transcription 9. Constitutive activation of ERK signaling drives oncogenesis by promoting uncontrolled cell proliferation, survival, and malignant progression 9. Knockdown of *KMT5B* reduces both phosphorylated and total ERK1 levels, thereby inhibiting ERK signaling activation 9. These findings underscore the role of KMT5B in driving SCCHN progression via ERK1 regulation, thereby positioning it as a promising therapeutic target for this malignancy.

### 3.4 Breast Cancer

The diagnosis and treatment of breast cancer have established an increasingly mature protocol in light of the rapid development of targeted therapy and immunotherapy and the rich research on breast cancer occurance and metastasis 66. While KMT5B is genomically amplified and classified as a key lysine methyltransferase in breast cancer cell lines 67, paradoxically, invasive subtypes demonstrate reduced KMT5B expression, with functional studies confirming that ectopic KMT5B expression elevates H4K20me3 levels and suppresses malignant invasion 68 (Figure 3A).

### 3.5 Hepatocellular Carcinoma

Hepatocellular carcinoma (HCC) maintains a high recurrence rate and poor prognosis despite radical surgical resection, with current multikinase inhibitor therapies demonstrating limited clinical efficacy 69. Emerging research on histone modifications, particularly KMT5B-mediated H4K20 methylation, has advanced understanding of HCC pathogenesis and therapeutics.

Elevated KMT5B expression is associated with aggressive features in HCC, including larger or multiple tumors, poor differentiation and vascular invasion, and independently predicts reduced 5-year survival 10. KMT5B promotes HCC by facilitating G1/S cell cycle progression and enhancing tumor proliferation 10 (Figure 3A). Overexpression of KMT5B is associated with poor prognosis in HCC, and inhibition of KMT5B could probably inhibit tumor progression and invasion.

### 3.6 Blood Cancer

KMT5B correlates with multiple blood cancers. Chronic myeloid leukemia (CML), characterized by BCR-ABL fusion in 95% of cases, remains difficult to treat in TKI-resistant patients 70. KMT5B promotes G1/S phase transition in CML K562 cells by suppressing p21 expression 7 (Figure 3A). Acute myeloid leukemia (AML) is a fatal bone marrow stem cell cancer 71. Genome-wide analyses reveal *KMT5B* as a key risk gene, demonstrating the role of histone methylation in leukemogenesis 72. These findings establish epigenetic dysregulation through KMT5B-mediated chromatin modification as a critical pathogenic mechanism, providing new insights into blood cancer heritable risk and highlighting potential therapeutic targets in histone modification pathways.

### 3.7 Lung Cancer

While targeted therapies significantly prolong survival in genetically-selected advanced non-small cell lung cancer (NSCLC) patients, their efficacy and clinical utility remain limited 73. A study on lung adenocarcinoma screened genes associated with disease-free survival in lung cancer by machine learning, and *KMT5B* was one of the top-ranked genes related to disease-free survival and negatively correlated with other outcomes 74 (Figure 3A).

## 4. KMT5B in Non-cancerous Diseases

### 4.1 Neurodevelopmental-Related Diseases

#### 4.1.1 Neurodevelopmental disorders (NDDs)

NDDs comprise a heterogeneous group of conditions unified by disrupted brain development, including autism spectrum disorder (ASD) and schizophrenia (SCZ) 75. Recent genetic studies have identified KMT5B as a key risk factor for ASD, and large-scale whole-genome sequencing across ASD cohorts supports its pathogenic role 15,52. *KMT5B* mutations, including pathogenic alternative splicing variants, missense mutations, frameshift deletions, and non-synonymous substitutions, consistently demonstrate LoF effects in ASD patients 15,52. *KMT5B* LoF mutations contributes to NDDs and ASD pathogenesis by disrupting DNA repair, increasing cellular stress, and impairing glutamatergic transmission in the prefrontal cortex, particularly during the critical postnatal period of circuit refinement 15. KMT5B exhibits dynamic subcellular localization patterns that include microtubule association in Xenopus laevis embryos and human neural cells, nuclear residence during interphase, mitotic spindle relocation during mitosis, and axonal microtubule targeting in differentiated neurons, suggesting its functional involvement in microtubule regulation 76. Cross-species studies in zebrafish, amphibians, rodents, and primates demonstrate the evolutionarily conserved role of KMT5B in neurogenesis, establishing H4K20 methylation as a key epigenetic regulator of normal neurodevelopment 18,52,57.

A prior study identified 78 genes with an excess of *de novo* mutations, including 32 novel NDD-associated genes like *KMT5B*, among 208 candidates screened in more than 11,730 patients 51. The study identified 4 additional KMT5B variants (2 LGD, 2 missense), with clinical data from 7 patients showing universal Intellectual Disability/Developmental Delay (ID/DD) (100%), high ASD prevalence (83%), and frequent speech/motor delays, seizures, or attention deficits 51. Another recent study identified three unrelated patients with *de novo* heterozygous *KMT5B* variants, presenting global developmental delay, intellectual disability, and macrocephaly 77. Three-dimensional (3D) modeling demonstrated that these variants affect hydrogen bonds, supporting *KMT5B*'s role in neurodevelopmental disorders with such features 77. Macrosomia represents a core feature, affecting 30 of 31 patients. Neuroimaging shows enlarged ventricles and vascular anomalies, suggesting that KMT5B regulates cerebrospinal fluid (CSF) dynamics 18.

Heterozygous *Kmt5b* deletion in mice results in a broad spectrum of neurodevelopmental and behavioral deficits, including impaired neonatal reflexes, abnormal social behavior, repetitive stress-induced grooming, altered pain perception, modified emotional responses, impaired fear extinction learning, and reduced body weight, length, and brain size 16. Sex-differential study demonstrates that *Kmt5b*-deficient mice exhibit significantly reduced body size from postnatal day 10 through juvenile development compared to wild-type littermates, correlating with microcephaly, with this phenotype showing greater severity in males than females 16. Delayed eye opening and weaker reflexes compared to wild-type littermates were observed in* Kmt5b*-deficient male neonates 16.

In human cerebral cortex organoid models, single-cell RNA-seq and proteomic profiling of more than 745,000 cells have been used to characterize phenotypic convergence caused by haploinsufficiency of three major ASD risk genes: *KMT5B*, *ARID1B*, and *CHD8* 14. All three mutations disrupt the coordinated development of cortical GABAergic interneurons and deep-layer excitatory projection neurons, despite acting through distinct molecular mechanisms 14.

These findings highlight the critical need for future circuit-level analyses to delineate shared pathophysiological mechanisms and inform therapeutic strategies.

#### 4.1.2 Craniosynostosis (CS)

CS, the most common congenital cranial disorder in humans, manifests as a syndromic form in ~15% of cases 78. Syndromic CS is defined by premature cranial suture fusion, severe facial dysmorphism, and limb anomalies, often resulting in increased intracranial pressure and neurodevelopmental delay 78. However, nearly 40% of syndromic CS cases remain without a genetic diagnosis at clinical evaluation 78.

A recent exome sequencing study of 526 syndromic CS proband-parent trios identified a significant excess of damaging DNVs in genes highly resistant to loss-of-function variation 78 (Figure 3B). Thirteen of these genes possessed excess DNVs and exceeded the threshold for genome-wide significance, with *KMT5B* being one of them 78. Three damaging DNVs in *KMT5B* were identified in CS patients, and *KMT5B* mutations underlie both syndromic and non-syndromic neurodevelopmental disorders 78 (Figure 3B).

### 4.2 Facioscapulohumeral Muscular Systrophy (FSHD)

FSHD, the third most common autosomal dominant myopathy, displays marked phenotypic heterogeneity 79,80. It typically begins with facial muscle involvement, which progresses asymmetrically to weakness in the shoulders and upper arms, followed by impairment of the trunk, pelvis, and lower limbs. Vascular anomalies and cognitive deficits are often concurrent 79,80. FRG1 binds KMT5B to induce dystrophic defects, and its overexpression or KMT5B depletion upregulates the myogenic repressor Eid3, forming an epigenetic FRG1/KMT5B-Eid3 axis that disrupts myogenic differentiation and contributes to FSHD pathogenesis 58 (Figure 3B).

### 4.3 PH/COPD

Pulmonary artery remodeling with smooth muscle cell proliferation occurs in mild COPD and may precede disease onset 81. Inactivation of KMT5B impairs postnatal lung development, induces COPD and pulmonary hypertension, and reduces H4K20me2/3 levels, supporting a pathogenic role for epigenetic dysregulation and highlighting KMT5B as a potential therapeutic target 49 (Figure 3B).

### 4.4 Meier-Gorlin Syndrome (MGS)

MGS, is a rare autosomal recessive primordial dwarfism disorder defined by the pathognomonic triad of microtia, patellar hypoplasia or agenesis, and short stature; ORC1 mutations have been identified as a major genetic cause 82-84. In patients with MGS, recurrent ORC1 mutations within the BAH domain, including F89S and E127G, disrupt H4K20me2 recognition 6. These findings suggest disrupted ORC1-H4K20me2 interactions in MGS pathogenesis and highlight the critical role of H4K20me2 in primitive dwarfism 6 (Figure 3B).

### 4.5 Obesity

Obesity is a global epidemic with elevated risks of hypertension, diabetes, and cancers 85. Cold exposure downregulates *KMT5B* and *KMT5C* in BAT, with combined knockdown eliminating H4K20me3 85. KMT5B/KMT5C suppress PPAR-γ, a master regulator of lipid/glucose metabolism 85. Mice lacking both proteins resist obesity via PPAR-γ target gene activation in BAT, enhancing mitochondrial respiration, glucose tolerance, and reducing adipose mass 85. Thus, KMT5B/KMT5C are epigenetic regulators of PPAR-γ pathways, serving as promising targets for metabolic disorders 85 (Figure 3B).

### 4.6 Friedreich's Ataxia (FRDA)

FRDA, the most common hereditary ataxia, is a neurodegenerative disorder characterized by neurologic dysfunction, cardiomyopathy, and diabetes, with no available treatment currently 86. It primarily arises from LoF mutations in the frataxin (*FXN*) gene on chromosome 9q13, with most patients harboring amplified GAA trinucleotide repeats in intron 1 of both FXN alleles. This repeat expansion reduces gene transcription and frataxin expression 86,87.

Recent study has identified KMT5B as a key regulator of *FXN* expression and a promising therapeutic target for FRDA 87. Using a human FXN-GAA-luciferase reporter model, pharmacological inhibition of KMT5B/5C methyltransferases with the compound A196 significantly increased FXN expression without affecting wild-type cells 87. This inhibition was accompanied by decreased H4K20me2/3 and increased H4K20me1 marks, while global gene expression remained largely unaltered 87. These findings underscore the critical role of histone methylation in regulating FXN and establish KMT5B as a validated therapeutic target for FRDA 87 (Figure 3B).

## 5. Emerging KMT5B Inhibitor A-196

A-196 is the first potent and highly selective inhibitor of KMT5B and KMT5C enzymatic activity 21. A-196 acts as a substrate-competitive inhibitor and exhibits positive cooperativity when binding to KMT5B in the presence of SAM, it directly binds to the histone H4 peptide-binding groove of the enzyme, thereby blocking the catalytic site 21. Identified through high-throughput screening and subsequent structural optimization, A-196 exerts potent inhibitory effects on SUV420H1 (with an IC₅₀ of 25±5 nM) and SUV420H2 (with an IC₅₀ of 144±21 nM) 21. Intracellular experiments have confirmed that A-196 induces a global increase in the level of H4K20me1 and a concomitant decrease in the levels of H4K20me2 and H4K20me3 in cells 21. This regulatory effect occurs across all phases of the cell cycle, and no obvious toxicity has been observed in multiple cell lines 21. Guided by the structure-activity relationship and crystal structure of A-196, a series of highly potent analogs were synthesized in a recent study 87. Among these derivatives, A3 and A12 exhibited markedly superior efficacy in upregulating FXN expression compared with the parent compound A-196; in particular, A3 had an EC₅₀ as low as 0.21 μM, representing a ~24-fold increase in potency relative to A-196, and could significantly elevate FXN mRNA levels in primary fibroblasts derived from FRDA patients 87. These findings thus provide novel directions and promising candidate molecules for the development of clinical drug candidates against FRDA.

Before the development of A-196, studies typically relied on techniques such as gene knockout and RNA interference to reduce the expression of KMT5B 1,3. These approaches exert irreversible effects and overlooked the non-catalytic functions of KMT5B. In contrast, A-196 enables reversible, rapid, and precise inhibition of KMT5B and KMT5C enzymatic activities, which facilitates to clarify its effect mechanism in diseases and helps the research on targeted drugs. However, A-196 has the limitation of failing to distinguish between KMT5B and KMT5C, making it impossible to rule out the interference caused by another enzyme. With A-196 as a reference, a study identified two potential KMT5B inhibitors (ZINC08398384 and ZINC08439608) through *in silico* simutation and *in vitro* biological experiments 22. When ZINC08398384 and ZINC08439608 were applied to human osteosarcoma U2OS cells, the cells exhibited proliferation inhibition, downregulation of H4K20me2, and reduced migration ability 22. However, this study did not detect the inhibitory activity of the compounds against other epigenetic targets (such as KMT5C) 22. Further research is still needed to clarify their specificity and potential for translation into clinical drugs.

## 6. Conclusions and Perspectives

Epigenetics, acting as a regulator of genes, is gaining widespread attention for its role in disease onset and progression, as well as for its potential as a therapeutic target. KMT5B is a crucial methyltransferase in epigenetic processes. Years of research have gradually elucidated its mechanism of binding to nucleosomes and performing methylation of H4K20me1. Additionally, non-catalytic functions of KMT5B have been discovered, including promoting nucleosome-DNA dissociation and chromatin condensation. Building on this foundation, KMT5B possesses multiple functions that help maintain genomic stability, including facilitating proper DNA replication initiation, suppressing abnormal transcription, and repairing DSBs.

However, in different diseases, KMT5B can exhibit two opposite states: overexpression and deficiency. For instance, in cancer, KMT5B cannot be uniformly classified as either a tumor suppressor or oncogene. In GBM and sarcomas, KMT5B loss correlates with poor prognosis, whereas in SCCHN and HCC, KMT5B overexpression is associated with poor outcomes—some underlying mechanisms remain experimentally unconfirmed. In numerous non-neoplastic diseases, KMT5B dysfunction frequently causes pathogenicity.

Overall, due to KMT5B's dual catalytic and non-catalytic roles, and its tissue-specific binding to distinct targets to maintain normal physiological functions, no KMT5B-targeting drugs have yet entered clinical trials. However, in basic experiments, A-196 has been shown to simultaneously inhibit the enzymatic activity of both KMT5B and KMT5C, while ZINC08398384 and ZINC08439608 demonstrate potential for specific inhibition of KMT5B. As future research further validates the structure and function of KMT5B, it is anticipated that the functional map of KMT5B in the human body will be refined, achieving precision therapy targeting KMT5B epigenetics.

## Abbreviations

H4K20: lysine 20 on histone h4
PTMs: post-translational modifications
CD: catalytic domain
SET domain: (Su(var)3-9, Enhancer-of-zeste, Trithorax) domain
DSB: double-strand break repair
ARM: arginine-rich motif
ORC: origin recognition complex
MCM: minichromosome maintenance
pre-RC: pre-replication complex
MEFs: mouse embryonic fibroblasts
ORIs: origins of replication
BAH: bromo-adjacent homology
MuSC: skeletal muscle stem cell
NHEJ: non-homologous end-joining
HR: homologous recombination
NSPCs: neural stem/progenitor cells
SVZ: subventricular zone
GBM: glioblastoma
fHC: facultative heterochromatin
MCCs: multiciliated cells
Mos: morpholino oligonucleotides
COPD: chronic obstructive pulmonary disease
PH: pulmonary hypertension
Sod3: superoxide dismutase 3
DIPG: diffuse endogenous pontine glioma
ECM: extracellular matrix
ERK: extracellular signal-regulated kinase
ASD: autism spectrum disorder
LoF: loss of function
DEGs: differentially expressed genes
Hsp: heat-shock proteins
HNSCC: head and neck squamous cell carcinoma
HCC: hepatocellular carcinoma
CML: chronic myeloid leukemia
AML: acute myeloid leukemia
NSCLC: non-small cell lung cancer
NDDs: neurodevelopmental disorders
SCZ: schizophrenia
ID/DD: intellectual disability/developmental delay
CSF: cerebrospinal fluid
CS: craniosynostosis
DNVs: *de novo* variants
FSHD: facioscapulohumeral muscular systrophy
MGS: meier-gorlin syndrome
FRDA: friedreich's ataxia FXN frataxin
